# Unveiling the impact of non-coding RNAs on virus-induced cellular autophagy: roles and research advances

**DOI:** 10.3389/fmicb.2025.1632425

**Published:** 2025-08-06

**Authors:** Ming Yu, Ying Yi, Honglin Yang, Yi Zhang

**Affiliations:** ^1^Institute of Zoonosis, College of Public Health, Zunyi Medical University, Zunyi, China; ^2^Key Laboratory of Maternal & Child Health and Exposure Science of Guizhou Higher Education Institutes, Zunyi, China; ^3^Qingdao Municipal Center for Disease Control and Prevention, Qingdao, China; ^4^China Animal Health and Epidemiology Center, Qingdao, China

**Keywords:** non-coding RNAs, autophagy, viral infection, regulatory mechanism, virology

## Abstract

Autophagy is the process by which cells degrade and recycle damaged organelles and macromolecules by forming autophagosomes. This process is closely related to the maintenance of cellular homeostasis, ontogeny, and the occurrence and development of various diseases. Non-coding RNAs (ncRNAs) are a class of RNA molecules that do not encode proteins but play crucial roles in regulating gene expression. Numerous studies have demonstrated that ncRNAs are involved in regulating autophagy, and accumulating scientific evidence suggests that ncRNAs play an essential role in virus-induced cellular autophagy. ncRNAs affect autophagy by participating in the autophagy regulatory network, mediating the transcriptional and post-transcriptional regulation of autophagy-related genes. This review aims to explore the role of ncRNAs in autophagy induced by viral infection and analyze the relevant molecular regulatory mechanisms underlying autophagy. By examining the content above, we speculate that targeted regulation of ncRNAs can affect autophagy induced upon viral infection, thereby achieving antiviral effects and host cell protection.

## Highlights

• ncRNAs play a key role in regulating viral infection-induced autophagy, influencing cellular homeostasis and viral replication via critical signaling pathways.
• ncRNAs can both promote and inhibit autophagy during viral infections, with effects varying by virus type and host cell context.
• Emerging research reveals ncRNAs’ dual role in autophagy and viral replication, offering potential targets for antiviral drug development.
• Targeted ncRNA regulation may provide novel therapeutic strategies to modulate autophagy, control viral infections, and protect host cells.

## 1 Introduction

Autophagy is recognized as a cyclic degradation mechanism that removes protein aggregates, damaged organelles, and intracellular pathogens to control cell damage. It is essential for the survival of eukaryotic cells and mammals, playing a key role in maintaining cellular homeostasis, development, tumorigenesis, and viral infections (Levine and Kroemer, 2009, 2019; Ghafouri-Fard et al., 2022). Autophagy is a complex cellular process that involves the delivery of cellular contents to lysosomes for degradation and the subsequent recycling of macromolecules formed from this degradation (Chang, 2020). The three primary forms of autophagy—microautophagy, macroautophagy, and chaperone-mediated autophagy—differ in their physiological functions and mechanisms of lysosomal delivery. Among these, macroautophagy is the most critical and widely studied form Wu et al. (2023). Macroautophagy engulfs cytoplasmic contents, including protein aggregates, damaged organelles, and intracellular pathogens, into double-membrane autophagosomes. These autophagosomes fuse with lysosomes to form autolysosomes, where their contents are degraded, and the resulting macromolecules are recycled for cellular reuse (Ueno and Komatsu, 2017). The autophagy process is regulated by a variety of cellular regulators, which play a crucial role in maintaining cellular homeostasis. Genetic or functional defects may lead to autophagy disorders, which in turn affect cell homeostasis and physiological balance *in vivo* (Füllgrabe et al., 2016; Lee, 2018; Gómez-Virgilio et al., 2022). In addition, autophagy defects can influence the pathogenesis of various diseases, and their abnormalities are associated with the onset and progression of multiple diseases. For example, the single allelic deletion of the autophagy-related gene Beclin-1 in various human cancers reduces autophagy activity, thereby increasing the risk of cancer (Li et al., 2010; Tang et al., 2015). Autophagy is crucial in viral infections, and defects in autophagy-related genes can affect the host’s antiviral immune response and viral lifecycle. For instance, the deletion of the autophagy-related gene ATG5 can increase interferon production against RNA viruses such as VSV, thereby inhibiting viral replication (Choi et al., 2018). In contrast, another study has shown that ATG5 deficiency can lead to mitochondrial dysfunction, induce a large amount of ROS synthesis, upregulate the RLR signaling pathway, and trigger the excessive secretion of IFN-α and IL-6, resulting in host cell damage (Kim et al., 2010). Moreover, viral infection can activate or inhibit autophagy in host cells, thereby affecting virus replication and spread. For example, infection with SARS-CoV-2 activates ULK-1-Atg13 and VPS34-VPS15-BECN1 pathways, promoting autophagosome formation and inhibiting SARS-CoV-2 replication (Zhou et al., 2023). It is worth noting that viruses have evolved mechanisms to combat autophagy. For example, SARS-CoV-2’s ORF3a inhibits autophagosome-lysosome fusion by blocking SNARE complex assembly, mediated by the HOPS complex, thereby reducing autophagic flux. This action enables SARS-CoV-2 to evade immune clearance, facilitating viral survival and replication within host cells and enhancing its pathogenicity (Miao et al., 2021). These studies suggest that autophagy acts as a double-edged sword in viral infection, either contributing to the antiviral response or possibly being exploited by the virus to evade the immune response and promote its replication.

Non-coding RNAs (ncRNAs) are RNA molecules that do not encode functional proteins or peptides (Matsui and Corey, 2017). However, ncRNAs are involved in numerous cellular activities, such as gene activation and silencing, RNA splicing, modification and editing, protein translation, and binding to chromatin modification complexes (Kim et al., 2025; Margvelani et al., 2025). They also play a role in the transcription of enhancer RNA, contributing to the dynamic regulation of gene expression and cellular functions (Liu Z. et al., 2023; Mattick et al., 2023; Zhang et al., 2024). The regulatory role of ncRNAs in viral infections is highly diverse. They can not only regulate viral protein expression by interacting with viral RNA or the host’s transcriptional machinery, but also modulate antiviral immunity by influencing the production and activity of interferons and other cytokines (Chen et al., 2023; Liu X. et al., 2023). In recent years, an accumulating body of evidence suggests that ncRNAs play a significant regulatory role in autophagy induced upon viral infection (Beermann et al., 2016). For instance, following adenovirus infection, IGFBPrP1 expression increases. This upregulation of IGFBPrP1 in turn boosts the expression of lncRNA NEAT1, which subsequently enhances autophagy in mouse hepatic stellate cells by modulating the miR-29b/Atg9a axis (Kong et al., 2019). Additionally, the expression of miR-193b-3p is markedly upregulated in patients with chronic hepatitis B after HBV infection. This miRNA enhances the Akt/MDM2/p53 signaling pathway by targeting IGF-1R, thereby promoting cell autophagy and enhancing the post-transcriptional activity of HBV (Deng et al., 2025). Moreover, circular RNA also plays a crucial role in regulating autophagy induced upon viral infection. circ-Vav3 can sponge gga-miR-375 to activate the CIP2A/AKT axis, thereby suppressing autophagy induced by avian leukosis virus subgroup J (ALV-J) infection (Chen et al., 2025). These findings have shed light on the complex interactions between viruses and host cellular processes, revealing potential targets for therapeutic intervention. This article reviews the regulatory effects and mechanisms of ncRNAs on autophagy induced upon viral infection. A deeper understanding of the relevant processes and mechanisms will contribute to the development of antiviral drugs and further basic research.

## 2 Regulatory network of autophagy and the mechanism of ncRNAs action

**FIGURE 1:**
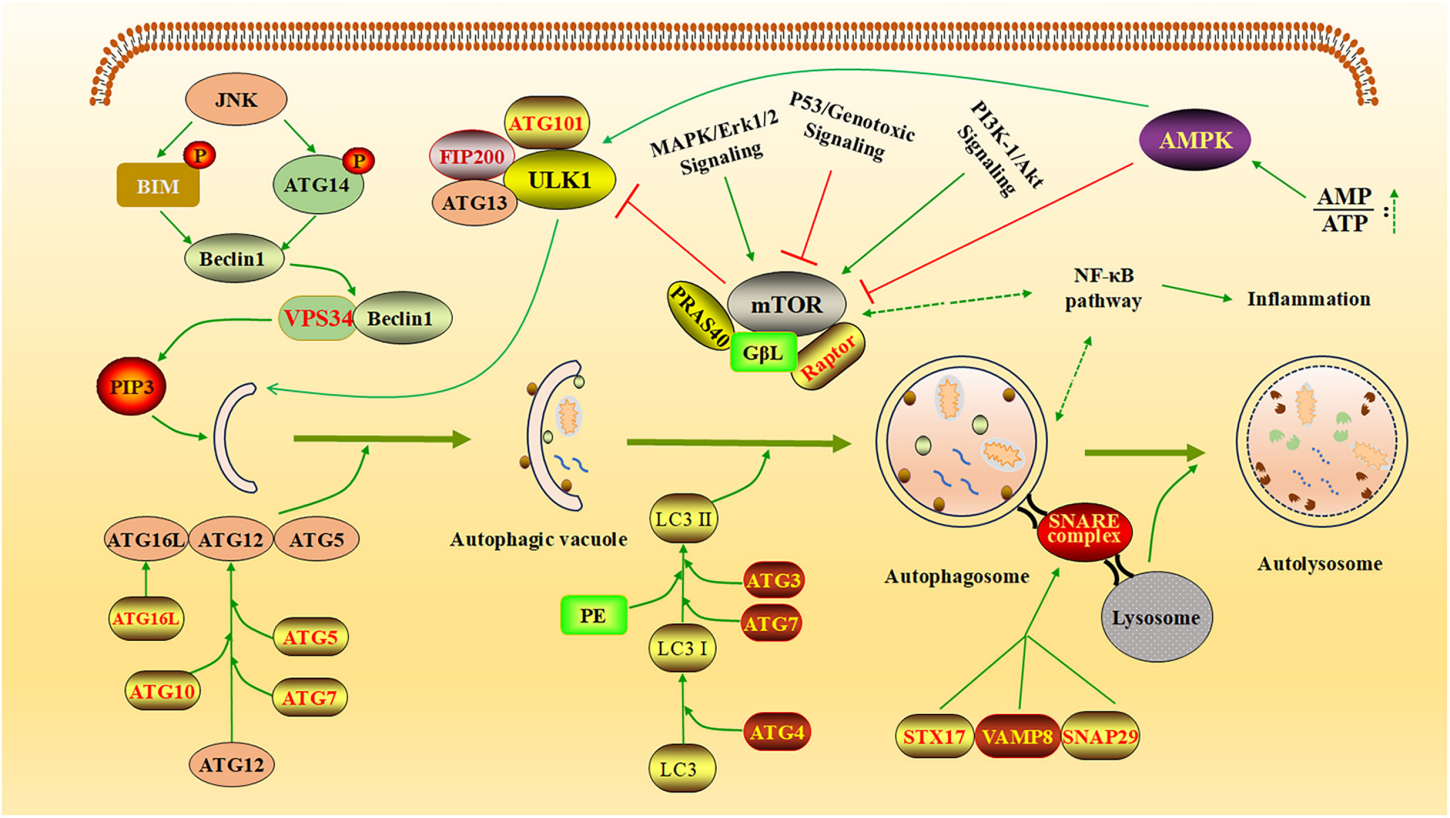
The regulatory network of autophagy. The diagram illustrates how autophagy is regulated by several key cellular pathways and protein complexes. AMPK inhibits the formation of the mTOR complex, thereby reducing mTOR’s suppressive effect on the ULK1 complex and promoting the generation of autophagic vesicles (Khandia et al., 2019). JNK kinase phosphorylates BCL-2 and BIM, leading to the release of Beclin-1. Free Beclin-1 activates VPS34 and binds to it, forming a complex that facilitates the production of PI3P, which promotes the elongation of autophagic vesicles. The autophagy process is further supported by two essential coupling mechanisms: the Atg12-Atg5-Atg16L system and the LC3/ATG8-PE conjugation system (Chen et al., 2023). These systems play critical roles in tagging specific proteins for autophagic degradation and actively participating in autophagosome formation. Additionally, STX17 binds to SNAP29 and VAMP8 to form the SNARE complex, which translocate to the autophagosome membrane, enabling the fusion of lysosomes with autophagosomes and the formation of autolysosomes (Liang et al., 2021). Created with BioRender.com and Microsoft PowerPoint 2019.

Autophagy is a highly intricate cellular process essential for maintaining homeostasis and responding to various stressors. It is tightly regulated by multiple vital signaling pathways and protein complexes (Figure 1).

Among these, the mTOR, AMPK, PI3K/Akt, and NF-κB pathways play pivotal roles in modulating autophagy activity, often responding to changes in cellular energy status, nutrient availability, and external stress signals (Alharbi et al., 2021; Park et al., 2023). The mTOR pathway acts as a central regulator of cell metabolism, suppressing autophagy initiation in response to nutrient sufficiency, such as high amino acid levels, to prioritize growth and biosynthesis (Panwar et al., 2023). The AMPK pathway functions as an energy sensor, activating autophagy under conditions of low energy (e.g., reduced ATP levels) to restore energy homeostasis and maintain cellular balance (Garcia and Shaw, 2017). The PI3K/Akt pathway indirectly inhibits autophagy by upregulating mTOR activity, promoting cell survival and metabolism in response to external signals, such as growth factors or insulin (Liu et al., 2020). The NF-κB pathway regulates autophagy through the transcriptional control of autophagy-related genes, particularly during conditions of external stress, such as inflammation or oxidative stress, to balance cell survival and stress responses (Zhong et al., 2024). These pathways work together to fine-tune autophagy activity, ensuring dynamic regulation in response to cellular and environmental changes. The process of autophagy involves a coordinated series of steps regulated by distinct protein complexes. The ULK1 complex initiates autophagy by integrating upstream signals and triggering the formation of autophagosomes (Kishi-Itakura et al., 2014). This is followed by phagophore nucleation, which is mediated by the VPS34 complex, a core component of the class III PI3K machinery. Autophagosome elongation and closure depend on the ATG12-ATG5-ATG16L1 complex, which facilitates membrane expansion (Lystad et al., 2019). Additionally, LC3 (microtubule-associated protein light chain 3), a widely recognized marker of autophagosomes, plays a crucial role in cargo sequestration by binding to membranes and autophagic receptors. Among these receptors, p62 (also known as SQSTM1) is particularly important for selective autophagy, as it recognizes and delivers ubiquitinated cargo to autophagosomes for degradation (Li et al., 2024). Therefore, influencing these cellular pathways and key proteins related to autophagy can regulate the autophagy process in host cells.

Non-coding RNAs are involved in regulating autophagy through various molecular pathways and essential protein complexes, thereby influencing the autophagic process. For example, miRNAs can regulate autophagy by influencing specific signaling pathways, such as the PI3K/AKT/mTOR pathway, or by targeting key proteins like LC3 (Gong et al., 2018). lncRNAs can interact with other biological macromolecules (DNA, RNA, and proteins) to regulate autophagy and cellular functions at multiple levels (Zhang et al., 2019; Statello et al., 2021). In addition, lncRNAs can also act as competing endogenous RNAs (ceRNAs) and indirectly affect the autophagy process by binding to miRNAs (Yang L. et al., 2017; Cao Z. et al., 2022). For example, the overexpression of lncRNA PTENP1 results in a significant increase in PTEN, which regulates autophagy through the PI3K/AKT signaling pathway. Simultaneously, PTENP1 can competitively bind to miRNA-17, miRNA-19b, and miRNA-20a, thereby promoting the expression of ULK1 and ATG7, and consequently facilitating cellular autophagy (Jung et al., 2010; Chen et al., 2015). Moreover, lncRNA APF promotes the expression of ATG7 by competitively binding to miR-188-3p, thereby facilitating cellular autophagy (Wang et al., 2015). Through a review of the published literature, we found that ncRNAs play a dual role in regulating autophagy during viral infections, functioning in both positive and negative modes of modulation. This, in turn, influences viral replication and the host’s cellular immune response.

## 3 The role of ncRNAs in promoting autophagy

**FIGURE 2:**
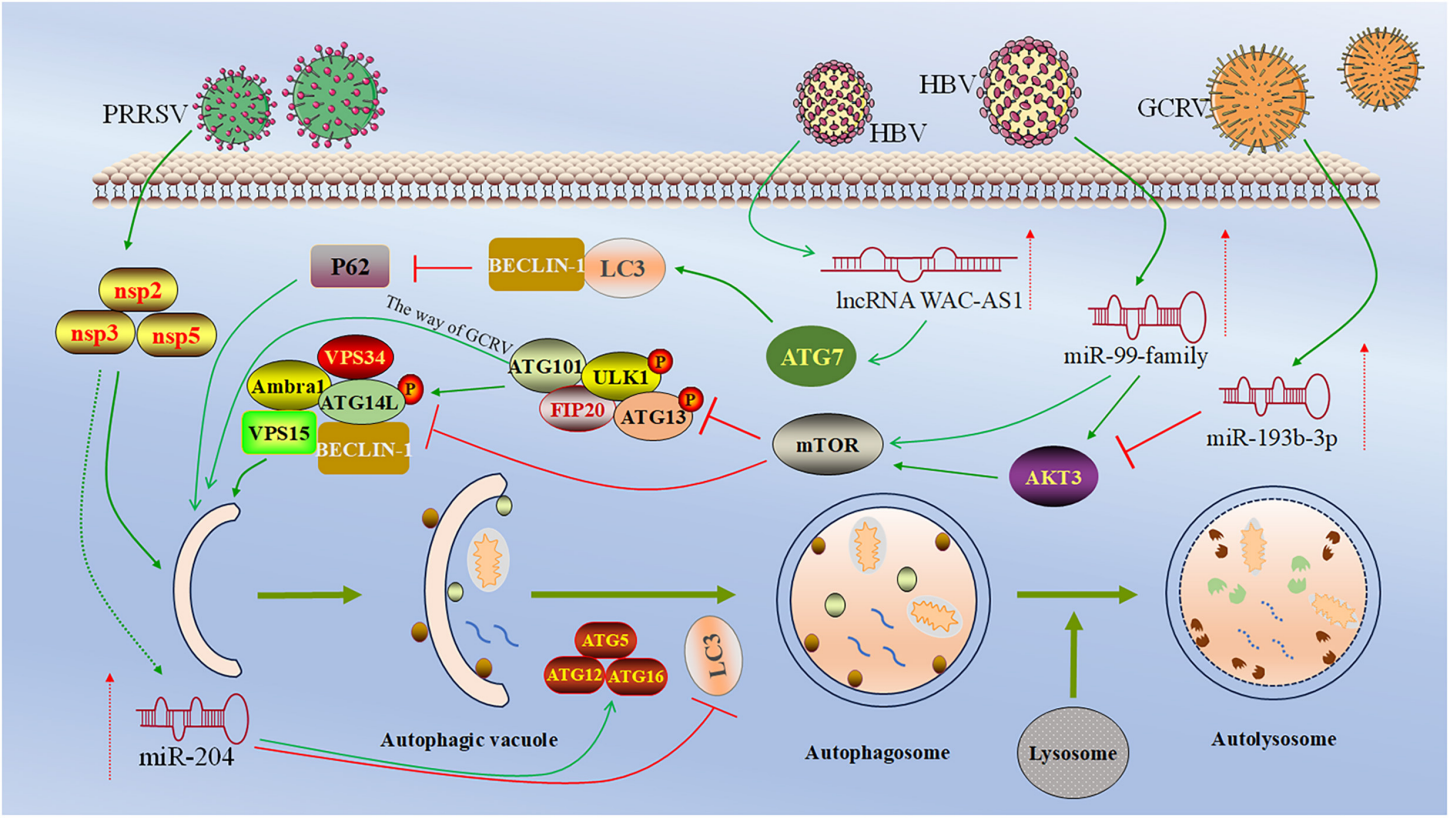
Regulatory roles and mechanism of ncRNA in viral infection-induced autophagy. This figure depicts the regulation of ncRNA in viral infection-induced autophagy. miR-99 family members target 3′-UTR of the mRNAs of IGF-1R, Akt, and mTOR, followed by the dephosphorylation of ULK1, leading to the initiation of autophagosome formation (Lin et al., 2017). miR-193b-3p targets the 3′-UTR of Akt3, inhibiting the expression of Akt3 and promoting autophagy in CIK cells (Yu et al., 2024a). miR-204 directly targets the 3′-UTR of LC3B and suppresses its expression, thereby inhibiting autophagy in PAMs cells (Yao et al., 2023). lncRNA WAC-AS1 enhances autophagy by targeting ATG7, enhancing LC3 and Beclin-1 expression and reducing p62 (Cao M. et al., 2022). Created with BioRender.com and Microsoft PowerPoint 2019.

Several studies have shown that miRNAs promote cell autophagy caused by HBV infection (Li et al., 2011; Lazar et al., 2014; Liu et al., 2014). For example, miR-99 family members can promote HBV self-replication by directly targeting the 3′UTR of IGF-1R, Akt, and mTOR mRNA, leading to ULK1 dephosphorylation to induce autophagy through the mTOR/ULK1 pathway (Figure 2; Lin et al., 2017).

Another study has shown that HBV core protein (HBc) and HBV X protein (HBx) upregulate the expression of miR-146a-5p through NF-κB in HBV-infected hepatocytes. This miRNA promotes HBV replication by targeting the Xiap-mediated MDM2/p53 axis, thereby inducing cell autophagy (Fu et al., 2019). In addition, Wang et al. (2019) found that the interaction between HBx and c-myc can inhibit the expression of miR-192-3p, and this miRNA can inhibit Xiap expression by targeting the 3′-UTR of Xiap, thereby affecting NF-κB. Therefore, HBV infection can promote autophagy and enhance HBV replication through the miR-192-3p/Xiap/NF-κB axis (Wang et al., 2019). It is worth noting that lncRNA also plays an essential role in HepG2.2.15 cells infected with HBV. For example, lncRNA WAC-as1 promotes autophagy of HepG2.2.15 cells by targeting ATG7 or enhancing the expression of miR-192-5p, thereby promoting HBV replication *in vitro* (Cao M. et al., 2022; Table 1). In conclusion, it is evident that ncRNAs play a role in inducing and promoting autophagy during HBV infection, and autophagy, in turn, encourages the replication of HBV both *in vivo* and *in vitro*.

**TABLE 1:** The role of non-coding RNAs in viral infection-induced autophagy.

Virus	Host species	ncRNA	Target gene	Cell type	Effects on autophagy	Effects on viral replication	Mechanisms on regulating autophagy	Reference
HBV	Human	miR-99 family	IGF1R/Akt/mTOR	Hepatoma cells	↑	↑	Targeting the 3′UTR of IGF-1R, etc. to affect the mTOR/ULK1 pathway	Lin et al., 2017
	Human	miR-146a-5p	Xiap	Hepatocytes	↑	↑	Targeting the Xiap-mediated MDM2/p53 axis	Fu et al., 2019
	Human	miR-192-3p	Xiap	Hepatocytes	↑	↑	Targeting the 3′-UTR of Xiap and inhibiting the expression of Xiap and affects NF-κB	Wang et al., 2019
	Human	lncRNA WAC-as1	miR-192-5p/ATG7	Hepatoma cells	↑	↑	Targeting ATG7 or enhancing miR-192-5p expression	Cao M. et al., 2022
	Human	miR-141	Sirt1	Hepatocytes	↓	↓	Targeting the 3′-UTR of Sirt1 and inhibiting the expression of Sirt1	Yang Y. et al., 2017
	Human	miR-193b-3p	IGF-1R	Hepatocytes	↑	↑	Targeting IGF-1R and enhancing the Akt/MDM2/p53 signaling pathway	Deng et al., 2025
HPV	Human	miR-155-5p	PDK1	Cervical cancer cells	↑	–	Targeted 3′-UTR of PDK1 and inhibition of Akt/mTOR signaling pathway	Wang et al., 2018
	Human	miR-224-3p	FIP200	Cervical cancer cells	↓	–	Targeting the 3′-UTR of FIP200 and inhibiting its expression	Fang et al., 2016
WSSV	Shrimp	miR-71	CAP-1	Shrimp hemocytes	↑	↑	Targeting the CAP-1 gene	He et al., 2017
	Shrimp	miR-13b	Knickkopf	Shrimp hemocytes	↑	↑	Targeting the knickkopf gene	He et al., 2017
PCV2	Pig	miR-30a-5p	14-3-3 gene	Porcine alveolar macrophages	↑	↑	Targeting 14-3-3 gene and promoting G2 phase cell cycle arrest	Wang et al., 2017
DEV	Duck	siRNA	AMPK/TSC2	Duck embryo fibroblast cells	↑	↑	Targeting AMPK or TSC2 to affect AMPK-TSC2-MTOR signaling pathway	Yin et al., 2017
PRRSV	Pig	miR-204	LC3B	Porcine alveolar macrophages	↓	↓	Targeting the 3′-UTR of LC3B and inhibiting its expression	Yao et al., 2023
RGNNV	*Lateolabrax japonicus*	miR-192	ULK-VAPs-Atg13	*Lateolabrax japonicus* brain cells	↓	↓	Targeting LjULK1 altered the distribution of LC3 and the expression of autophagy-related proteins	Pan et al., 2024
	*Lateolabrax japonicus*	miR-731	ULK-VAPs-Atg13	*Lateolabrax japonicus* brain cells	↓	↓		Pan et al., 2024
	Sea perch	lja-miR-145	–	*Lateolabrax japonicus* brain cells	↑	↑	Acting on LC3B-II/I and p62	Jia et al., 2021
	Sea perch	lja-miR-183	–	*Lateolabrax japonicus* brain cells	↓	↓	Acting on LC3B-II/I and p62	Jia et al., 2021
GCRV	*Ctenopharyngodon idella*	miR-193 b-3p	Akt3	Kidney cells of *Ctenopharyngodon idella*	↑	↓	Targeting the 3′-UTR of Akt3 to inhibit its expression	Yu et al., 2024a
	*Ctenopharyngodon idella*	miR-193b-5p	Detor	Kidney cells of *Ctenopharyngodon idella*	↓	↑	Targeting the 3′-UTR of deptor and inhibiting its mRNA expression	Yu et al., 2024b
BVDV	Bovine	miR-2904	ATG13	Madin-Darby bovine kidney cells	↓	↓	Targeting the 3′-UTR region of ATG13 mRNA	Yang et al., 2022
ARV	Chicken	gga-miR-30c-5p	ATG5	Chick embryo fibroblasts – DF-1	↓	↓	Targeting the 3′-UTR of ATG5 negatively regulating its expression	Zhou et al., 2022
ALV-J	Chicken	circ-Vav3	gga-miR-375/CIP2A/AKT	Chick embryo fibroblasts – DF-1	↓	–	By functioning as a sponge for gga-miR-375 and activating the CIP2A/AKT axis	Chen et al., 2025
DENV	Human	miR-146a	TRAF6	Human lung carcinoma epithelial cells and human monocytic cells	↓	–	Targeting TRAF6 and inhibiting IFN-β expression	Pu et al., 2017
EV71	Human	miR-30a	Beclin-1	Human epidermoid carcinoma and African green monkey kidney cells	↑	↑	Targeting the 3′-UTR of Beclin-1	Fu et al., 2015
RSV	Human	miR-136	Sirt1	Human bronchial epithelial cells	↓	↓	Activating Sirt1 signaling pathway	Wang et al., 2024
IAV	Human	lncRNA PCBP1-AS1	ATG7	Human lung epithelial cells	↑	↑	Acting on ATG7 promoter, resulting in upregulation of ATG7 expression	Chi et al., 2024
PEDV	Pig	siRNA	TAK1	African green monkey kidney cells and porcine intestinal epithelial cells	↑	↑	Downregulating TAK1, which in turn affected the AMPK/c-Jun pathway	Wang et al., 2022
ADV	Mouse	lncRNA NEAT1	miR-29b/Atg9a	Mouse hepatic stellate cells	↑	–	By sponging miR-29b and upregulating Atg9a expression	Kong et al., 2019
ZIKV	Drosophila	siRNA	–	Mosquito larval cells	↓	–	SNX5 or SNX32 proteins were silenced by siRNA	Liu et al., 2018
WNV	Human and mosquito			Human neuroblastoma cells				Kobayashi et al., 2014
CHIKV	Human and mouse			Human fibroblasts cells				Joubert et al., 2012
Poliovirus	Human			Human cervical cancer cells				Jackson et al., 2005
CVB3	Human and mouse			Pancreatic acinar cell				Alirezaei et al., 2012
IAV	Human			Human lung epithelial cells				Gannagé et al., 2009

“–” indicates not mentioned in the references.

The expression of miR-155-5p is decreased after HPV infection in cervical cancer cell lines. This miRNA inhibits the Akt/mTOR signaling pathway and enhances autophagy by targeting PDK1 expression (Wang et al., 2018). In addition, Enterovirus 71 (EV71) infection in Hep2 and Vero cells decreased the expression of miR-30a, which could enhance EV71 replication by targeting the 3′-UTR of Beclin-1 (a key autophagy-promoting gene) to induce autophagy (Fu et al., 2015). lncRNAs can affect autophagy induced upon influenza infection. For example, Chi et al. (2024) found that PESP, a small protein encoded by the lncRNA PCBP1-AS1, causes the upregulation of ATG7 expression by targeting the enhancement of ATG7 promoter activity, which promotes autophagy and leads to enhanced replication of IAV.

Non-coding RNAs can also enhance autophagy during animal virus infection. When porcine circovirus type 2 (PCV2) infected 3D4/21 cells, miR-30a-5p enhanced PCV2 virus-induced autophagy and promoted PCV2 replication by targeting the 14-3-3 gene, a regulator of autophagy that also promotes G2 phase cell cycle arrest (Wang et al., 2017). miRNAs can affect autophagy induced by white spot syndrome virus (WSSV) through different targets. For example, the expression of miR-71 is upregulated after WSSV infection, which promotes host autophagy by targeting the host calcification-associated peptide-1 (CAP-1) gene. Furthermore, host autophagy enhances the expression of miR-71. This eventually leads to improved WSSV replication (He et al., 2017). Similarly, miR-13b promotes autophagy caused by WSSV infection by targeting the host knickkopf gene, thereby facilitating the replication of WSSV. In addition, Jia et al. (2021) demonstrated that lja-miR-145 promoted red spotted grouper nervous necrosis virus (RGNNV)-induced autophagy in LJB cells and enhanced virus replication by acting on LC3B-II/I and p62 proteins. In addition, the expression of miR-193b-3p was increased in CIK cells infected with grass carp reovirus (GCRV). This miRNA targets the 3′-UTR of Akt3 (a key regulator of autophagy), inhibiting the expression of Akt3 and promoting autophagy in CIK cells and inhibiting the replication of the virus (Yu et al., 2024a).

Recent studies have demonstrated that siRNAs can modulate virus-induced autophagy by targeting and silencing specific proteins. Following infection with duck enteritis virus (DEV), siRNA can regulate autophagy by targeting AMPK or TSC2, thereby affecting the AMPK-TSC2-MTOR signaling pathway. In this process, autophagy can promote the replication of the DEV virus (Yin et al., 2017). When porcine epidemic diarrhea virus (PEDV) infects Vero cells, siRNA effectively downregulates the expression of TAK1, thereby affecting the AMPK/c-Jun pathway, promoting autophagy in Vero cells, and enhancing the replication of PEDV (Wang et al., 2022).

In both human and animal viral infections, ncRNAs play a role in enhancing autophagy within host cells. Our analysis reveals that ncRNAs can target host genes to promote autophagy, which, in turn, influences viral replication. It is worth noting that different ncRNAs can regulate autophagy induced upon the same virus. As mentioned above, miR-99 family, miR-146a-5p, miR-192-3p, and lncRNA WAC-as1 can all regulate HBV-induced autophagy. However, it remains uncertain whether these ncRNAs have synergistic effects that merit further exploration.

## 4 The role of non-coding RNAs in inhibiting autophagy

Non-coding RNAs can also act as negative regulators of autophagy. For example, miR-141 downregulated the expression level of Sirt1 after HBV infection in hepatocytes. This miRNA can directly target the 3′-UTR of Sirt1, thereby reducing the expression level of Sirt1 and inhibiting autophagy in hepatocytes. In turn, miR-141 can reduce the expression of HBV-DNA, HBsAg, and HBeAg, thereby reducing HBV replication, and may be developed into an RNA-based drug for HBV therapy (Yang Y. et al., 2017). In addition, Pu et al. (2017) demonstrated that miR-146a inhibited autophagy in dengue virus (DENV)-infected A549 and THP-1 cells by targeting TRAF6, resulting in decreased IFN-β expression. Through the regulation of autophagy, DENV2-induced TNF-α and IL-6 proinflammatory cytokine synthesis was enhanced. It also inhibited the excessive inflammation in host cells, thereby alleviating the immune damage caused by DENV2 infection (Pu et al., 2017). Additionally, FIP200 is a protein required for autophagosome formation by interacting with ULK1. HPV infection of cervical cancer cells can induce the upregulation of miR-224-3p. This miRNA can directly target the 3′-UTR of FIP200, thereby inhibiting FIP200 expression and subsequently suppressing autophagy in cervical cancer cells (Fang et al., 2016). Alternatively, the expression of miR-136 was upregulated in respiratory syncytial virus (RSV)-infected BEAS-2B cells. This miRNA inhibited cell autophagy by targeting the Sirt1 signaling pathway. Urolithin A can inhibit miR-136, thereby indirectly promoting cell autophagy and playing an antiviral role (Wang et al., 2024). Furthermore, silencing SNX5 or SNX32 proteins by siRNA has been shown to inhibit autophagy induced upon a variety of viruses, including Zika virus (ZIKV) (Liu et al., 2018), West Nile virus (WNV) (Kobayashi et al., 2014), chikungunya virus (CHIKV) (Joubert et al., 2012), poliovirus (Jackson et al., 2005), Coxsackievirus B3 (CVB3) (Alirezaei et al., 2012), and IAV (Gannagé et al., 2009).

During animal virus infections, accumulating evidence has shown that miRNAs can suppress viral replication by inhibiting cellular autophagy. For example, when porcine reproductive and respiratory syndrome virus (PRRSV) infects porcine alveolar macrophages (PAMs), miR-204 inhibits autophagy in PAMs cells. It reduces the level of PRRSV replication by directly targeting the 3′-UTR of LC3B and inhibiting its expression (Yao et al., 2023). Additionally, miR-192 and miR-731 target the 3′-UTR of LjULK1 in the ULK-VAPs-Atg13 pathway, altering the distribution of LC3 and the expression of autophagy-related proteins, thereby reducing the level of autophagy induced by RGNNV and inhibiting RGNNV proliferation (Pan et al., 2024). In another study, when RGNNV infects LJB cells, lja-miR-183 inhibits autophagy in LJB cells induced by RGNNV by targeting LC3B-II/I and p62, thereby reducing the replication levels of RGNNV (Jia et al., 2021). In bovine viruses, miR-2904 inhibits MDBK cell autophagy by targeting the 3′-UTR region of autophagy-related gene 13 (ATG13) mRNA during the infection of bovine viral diarrhea virus (BVDV). Overexpression of miR-2904 inhibits the replication of BVDV (Yang et al., 2022). In addition, avian reovirus (ARV) infection in DF-1 cells enhances the expression of gga-miR-30c-5p. This miRNA negatively regulates the expression of autophagy-related gene 5 (ATG5) by targeting its 3′-UTR, thereby inhibiting virus-induced autophagy in DF-1 cells. This inhibition of autophagy simultaneously suppresses ARV replication and syncytium formation, exerting an antiviral effect (Zhou et al., 2022). It is worth noting that ncRNAs can also promote viral replication by negatively regulating autophagy. For example, deptor, a protein containing the DEP domain that interacts with mTOR, downregulating deptor can inhibit cellular autophagy (Kim et al., 2011; Catena and Fanciulli, 2017). miR-193b-5p negatively regulates the expression of deptor mRNA by targeting the 3′-UTR of deptor. It reduces the level of autophagy in CIK cells and promotes the replication of GCRV virus (Yu et al., 2024b).

Above all, miRNAs play a critical role in the negative regulation of autophagy. These ncRNAs inhibit cellular autophagy by targeting key regulatory proteins or protein complexes, such as LC3B and ULK-VAPs-Atg13. Based on the above review, we speculate that targeted intervention of ncRNAs can affect autophagy induced by viral infection, thereby achieving antiviral effects and host cell protection.

## 5 Summary and prospects

In this study, we comprehensively reviewed the roles of ncRNAs in autophagy induced upon viral infections. It was demonstrated that ncRNAs play a key regulatory role in regulating cellular autophagy during viral infections. ncRNAs can promote or inhibit virus-induced autophagy by influencing crucial signaling pathways, such as IGF-1/PI3K/Akt/mTOR and AMPK/Erk/mTOR/ULK1, or by targeting key regulatory proteins, such as ATG5, Beclin-1, and LC3B. Therefore, ncRNAs-mediated post-transcriptional regulation may shed new light on the interplay between viral infection and host autophagy and may provide potential targets for drug development.

In addition, we found that ncRNAs affect autophagy as well as viral replication. For example, ncRNAs such as the miR-99 family, miR-146a-5p, and miR-192-3p promote autophagy induced by HBV, and also enhance HBV replication and expression. It is worth noting that the same ncRNA can exert distinct effects on autophagy regulation during viral infections. For instance, miR-146a promotes autophagy in HBV infection by targeting the Xiap-mediated MDM2/p53 axis, thereby facilitating viral replication. Conversely, during DENV infection, miR-146a inhibits autophagy by targeting TRAF6, thereby suppressing viral replication and reducing the production of proinflammatory cytokines. With a deeper understanding of ncRNAs’ function and mechanism, we can design specific drugs to modulate ncRNAs expression, thereby intervening in the autophagy process, affecting viral replication, and protecting host cells.

The regulatory network of ncRNAs is complex, involving a variety of molecules and signaling pathways. Understanding how these networks work in coordination across different cell types and physiological states is also an important direction for future research. Although we have some understanding of the role of ncRNAs in virus-induced autophagy, many mysteries remain. Future research should thoroughly investigate the interaction mechanisms between viral and host ncRNAs, elucidating how viruses precisely regulate host autophagy to facilitate their own replication and spread. Meanwhile, advanced techniques should be employed to investigate the dynamic regulatory roles of ncRNAs across different stages of autophagy. It is also crucial to focus on how ncRNAs influence viral drug resistance and develop novel antiviral therapies. Additionally, validating the potential of ncRNAs as biomarkers and therapeutic targets, as well as studying their specific functions in diverse cells and tissues, is of great importance. These research directions will enhance our understanding of ncRNAs in virus-related autophagy and drive the development of antiviral treatment strategies.
